# 

**Published:** 1896-07-11

**Authors:** 


**Notices of Births, Deaths, and Marriages** are inserted in this column at a charge of 2s. 6d. for an announcement not exceeding four lines.

**Notice to Advertisers and Subscribers.**

All Advertisements, Orders for Copies of The Hospital and Business Communications should be addressed to **THE MANAGER, (Not to The Editor),** The Hospital, 428, Strand, London, W.C.

The Cost of Subscription, post free, is as follows:—Per week, 2½d.; per quarter, 2s. 8d.; per half-year, 5s. 3d.; per annum, 10s. 6d. Foreign:—Per Annum, 15s. 2d.; payable, in all cases, in advance.

Just Published.

**"Burdett's Hospitals and Charities, 1896."**

Seventh year of publication. Over 1,000 pp. Scarlet cloth, gilt lettered, Price 5s. A most complete guide to British, Colonial, Indian, and American Hospitals, Dispensaries, Nursing and Convalescent Institutions, and Asylums. A few complete sets of the preceding six issues of the "Annual" may still be obtained on application to the Publishers, The Scientific Press (Limited), 428, Strand, London, W.C.

### NOTICE TO CORRESPONDENTS.

All MS., Letters, Books for Review, and other matters intended for the Editor should be addressed The Editor, The Lodge, Porchester Square, London, W.

The Editor cannot undertake to return rejected MS., even when accompanied by stamped directed envelope.

**NOTICE.—Vol XIX. of "The Hospital" (October, 1895, to April, 1896), in handsome cloth binding, is now on sale at the Office of this Journal. Price 7s. 6d. Cases for binding the Numbers contained in this Volume. 1s. 6d. Index to Vol. XIX., 2½d., post free. The Monthly Part for July is now ready, price 6d., by post 8d.**

### SCRAPS AND GLEANINGS.

A HOSPITAL is to be built at Aberdeen, New Glasgow, Canada.

A LEGACY of £2,000 has recently been left to the Salford Royal Hospital.

A COTTAGE hospital is to be built at Denny Co., Stirling. Over £1,000 has already been subscribed.

THE construction of the extension of the Monkwearmouth Hospital is being steadily proceeded with.

MR. C. T. GASCOIGNE has given the handsome sum of £4,000 to the Royal Hospital for Incurables, Dublin.

ABERDEEN has now instituted a Hospital Saturday movement, the first collection taking place during last month.

THE annual sale of the working party in connection with that institution contributed £103 to the funds of the Norwood Cottage Hospital.

ADDITIONAL wards for cases requiring isolation are to be erected at the Essex and Chelmsford Infirmary, and it is estimated they will cost £1,000.

THE University Medical College of Kansas City, Mo., propose to erect a new building in Twelfth and Harrison Streets, at an estimated cost of 50,000 dols.

A TERRACE has been constructed on the south-west and west sides of the Alexandra Hospital, Woodhall Spa, at a cost of about £200, of which £100 still remains to be raised.

NEARLY 6,000 cases of insanitation were reported to the local authorities during 1895 as the outcome of the labours of the Mansion House Council on the Dwellings of the Poor.

IT has been decided to name the accident ward at the Swansea Hospital the "Studt" ward, in recognition of the large sums collected for the hospital by Mr. Studt during the last three years.

THE plans for the proposed gynæcological department for the Edinburgh Royal Infirmary, which is to be erected on the site of the old Children's Hospital, have been approved generally by the managers.

ONE HUNDRED AND EIGHT males and 59 females were treated as in-patients at the Queenstown General Hospital during the past year. The expenditure amounted to £517, against £483 for the previous 12 months.

A COMMITTEE has been appointed to carry out an agreement for the sale of the old building of the Royal Alexandra Hospital at Rhyl to the Urban District Council for £3,500, and to enter into contracts for the erection of a new hospital.

THE Islington Guardians have agreed to purchase from the Metropolitan Asylums Board the small-pox hospital at Highgate Hill for £52,500. The hospital, which stands on nine acres of ground, is to be converted into an infirmary.

THE in-patients treated at the Beckett Hospital, Barnsley, during the year 1895-6 amounted to 2,116, and the out-patients to 405. Eighty of the in-patients were cases of colliery accidents who remained in the hospital 2,210 days, and cost £273.

THE cost of building the new cottage hospital for Tavistock and neighbourhood has been paid out of a legacy left to the institution by Miss Herring, whose family had been connected with the town for many years. The site was given by the Duke of Bedford.

IT is hoped that the working men and the ministers of the neighbourhood will this year organise collections in aid of the Louth Hospital and Dispensary. Some years ago a Hospital Saturday Fund was started, but during the last two or three years the movement has been allowed to collapse.

INCLUDING the new hospitals being and to be built, namely, the Brook Hospital at Shooter's Hill, the Park Hospital at Hither Green, and the Grove Hospital, the Metropolitan Asylums Board hope to have by the summer of 1898 accommodation for at least 5,400 fever and diphtheria patients.

THE total expenditure of the Metropolitan Asylums Board for the year ended October 5th, 1895, amounted to £553,976, of which £115 020 was spent on the maintenance of patients, £55,077 on repayment of loans, and £46,313 on interest on loans. The daily cost of maintenance varied from $6\frac{13}{32}$d. to 1s. $3\frac{6}{32}$d.

THE *Western Morning News* points out that out of about 160 Church collections made during the last year in aid of the Devon and Exeter Hospital "only four apparently come from sources other than those of the Established Church. Out of an income under this head of £744 8s. 9d. the Nonconformist bodies among them contribute £16 12s. 11d."

IT was stated at a meeting in connection with the annual demonstration by the Friendly and Trades' Societies on behalf of the funds of the Stockton and Thornaby Hospital that last year out of a total subscription of £2,156 the Stockton workmen subscribed no less than £1,530. During the last eleven years the workmen's annual contribution has averaged £1,460

THE condition of the river at Cambridge will soon be improved, as the town is being sewered on the separate system, and in future rain water only will be carried to the river. The governing bodies of Trinity College and Trinity Hall have instructed Mr. Chas. E. Gritton, A.M.I.C.E., M.S.A., of Westminster and Selhurst, to prepare plans and reports for draining those colleges, and to obtain tenders for the works.

THE Wimbledon Cottage Hospital was closed for about six weeks during the year 1895, one of the causes being the failure of water during the severe frost in February. Nevertheless, the number of patients treated during the year was, with one exception, the highest yet recorded. Altogether, 117 in and 457 out-patients were treated, the average cost per patient per day being 3s. 8d., and the average cost per patient being £3 6s. 9d.

IN a recent action brought by Liebig's Extract of Meat Company (Limited) to restrain the Chemists' Co-operative Society (Limited) and others from selling meat extract in wrappers similar to those used by the plaintiffs, and from passing off meat extract sold by the defendants as meat extract sold by the plaintiffs, Mr. Justice Kekewich ruled that the plaintiffs were entitled to an injunction on both grounds; also to damages and delivery up of the wrappers in the defendants' possession, and costs.

### BOOKS RECEIVED.

THOS. MURLEY.

"Skertchley's Physical Geography." Revised to date. By John H. Howell, B.A.

MAW, SON, AND THOMPSON.

"The Nurses' Handbook and Catalogue of Nursing Requisites."

MARCUS WARD AND CO. (LIMITED).

"Merry Thoughts: A Birthday Book, with Selections from Humorous Writers."

BLACKWOOD AND SONS.

"Fellow Travellers." By Graham Travers.

BALLIERE, TINDALL, AND COX.

"The Modern Treatment of Stone in the Bladder by Litholapaxy." By P J. Freyer, M.A., M.D., M.Ch.

"Chemical Notes and Equations." By G. H. Gemmell, F.I.C., F.C.S.

**Periodicals and Pamphlets.**—New York Medical Journal, Therapist, Clinical Journal, London, Literary Digest, Medical Pioneer Guy's Hospital Gazette, Scalpel, Medical Week, Charity Organisation Review, Pediatrics, Corpuscle, Industries and Iron, Electrical Review, Medical Press, Sunday at Home, Boy's Own Paper, Girl's Own Paper, Leisure Hour, Cornhill Magazine, Columbus Medical Journal, Public Health, Planter, Invention, Economic, Medical Times, Sun, Woman's Signal, Pearson's Magazine, Pears' Pictorial, Indian Medico-Chirurgical Review, Cassell's Family Magazine, Medical Bulletin, Verulam Review, Westminster Review, Windsor Magazine, Proceedings of the American Medico-Psychological Association at the Fifty-first Annual Meeting, held in Denver, June 11th—13th, 1895.

### THE STUDENT OF MEDICINE.

### VACANCIES.

The following vacancies are announced this week, the amount of salary in each case being given after the name of the institution:—

### Resident Medical Officers.

Brecon Infirmary.—Resident House Surgeon; unmarried; doubly qualified. Salary £70 annum, with furnished apartments, board, attendance, fire, and gas. Applications to W. Porell Price, Secretary, No. 6, Bulwark, Brecon, South Wales, by July 16th.

Chesterfield and North Derbyshire Hospital and Dispensary, Chesterfield,—Junior House Surgeon and Dispenser. Salary, £50 per annum, with board, apartments, and laundress. Applications to the Secretary by July 16th.

General Infirmary at Gloucester and the Gloucestershire Eye Institution.—Assistant House Surgeon, doubly qualified. Appointment for six months, but eligible for re-election. Board, residence, and washing provided, but no salary. Applications to the Secretary by July 15th.

Great Northern Central Hospital, Holloway Road, N.—House Surgeon. Salary £60 per annum, with board, lodging, and laundry in the hospital. Junior House Surgeon. Appointment for six months, with board, lodging, and laundry provided in the hospital. Applications on forms of application provided to be sent to the Secretary by July 20th.

Hospital for Consumption and Diseases of the Chest, Brompton.—Resident House Physician. Applications to the Secretary, by July 21st.

Newport and Monmouthshire Hospital.—House Surgeon; doubly qualified. Salary, £100 per annum, with board and residence, no stimulants provided. Applications to the Secretary by July 11th.

Isle of Wight Asylum.—Assistant Medical Officer, doubly qualified. Salary £100 per annum, with board, lodging, &c., but without alcohol. Applications to the Medical Superintendent, Whitecroft, Newport, Isle of Wight, by July 20th.

Joint Counties Asylum, Abergavenny.—Junior Assistant Medical Officer. Salary £100 per annum, increasing by two yearly instalments of £25 to £150, with board, lodging, and washing. Applications to the Medical Superintendent by July 18th.

Sheffield General Infirmary.—Junior Assistant House Surgeon; doubly qualified. Salary £50 per annum, with board and washing. Appointments for three years, but eligible for re-election. The offices of House Surgeon (salary £120), House Physician at £80, and Senior Assistant House Surgeon, at £60 per annum, are also vacant. Applications, to be addressed to the "Medical Staff of the Sheffield General Infirmary," to the care of the Secretary, by July 16th.

Taunton and Somerset Hospital.—House Surgeon; doubly qualified. Must enter into an engagement for not less than three years in case the Governors shall so long require his services. Salary £100 per annum, with board, lodging, and washing. Applications to J. H. Biddulph Pinchard, Secretary, 13, Hammet Street, Taunton, by July 13th.

### Non-Resident Medical Officers.

Paddington Green Children's Hospital, W.—Ophthalmic Surgeon; must be F.R.C.S.Eng. Applications to the Secretary by July 11th.

University of Aberdeen.—University Assistant in Physiology. Salary £150 per annum, rising by annual increments of £25 to £200. Applications to Professor MacWilliam, by July 13th.

### Medical Appointments.

**METROPOLITAN ASYLUMS BOARD.**

**METROPOLITAN ASYLUMS BOARD.**

**GREAT NORTHERN CENTRAL HOSPITAL,**
**HOLLOWAY ROAD, N.**

